# Inheritance and memory of stress-induced epigenome change: roles played by the ATF-2 family of transcription factors

**DOI:** 10.1111/j.1365-2443.2012.01587.x

**Published:** 2012-04

**Authors:** Ki-Hyeon Seong, Toshio Maekawa, Shunsuke Ishii

**Affiliations:** Laboratory of Molecular Genetics, RIKEN Tsukuba Institute3-1-1 Koyadai, Tsukuba, Ibaraki 305-0074, Japan

## Abstract

Data on the inheritance-of-stress effect have been accumulating and some mechanistic insights, such as epigenetic regulation, have also been suggested. In particular, the modern view of Lamarckian inheritance appears to be affected by the finding that stress-induced epigenetic changes can be inherited. This review summarizes the current data on the inheritance of stress effect and possible mechanisms involved in this process. In particular, we focus on the stress-induced epigenetic changes mediated by the ATF-2 family of transcription factors.

## Introduction: Lamarckian inheritance revisited

In his book ‘Philosophie Zoologique’, Lamarck speculated on a mechanism of evolution in which the use of a particular organ leads to its functional improvement. In Lamarck’s theory, this improvement is transmitted to subsequent generations, as shown in the famous example of the giraffe’s neck (1809). At that time, it was believed that species are gradually transformed into other species and that organisms have the innate capacity to progress through the generations. This hypothesis is referred to as the ‘inheritance of acquired characteristics,’ although, unfortunately, there is no known mechanism by which the acquired characteristics can be converted into genetic information. However, it is worth noting that Lamarck focused on adaptive and useful characteristics, rather than acquired traits.

**Figure 1:**
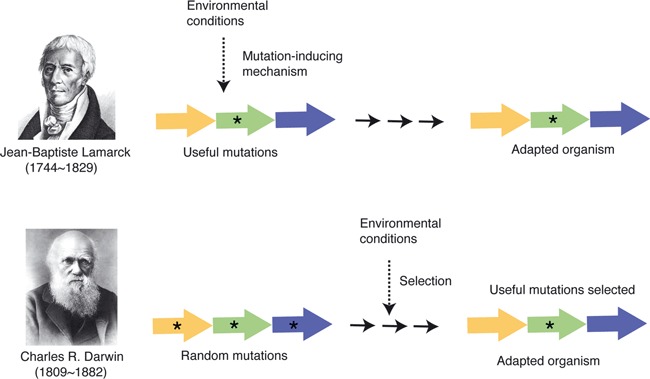
Comparison of Lamarckian and Darwinian evolution.

If Lamarck’s concept is modified using modern genetic terminology, it contains the following two key points: (i) environmental conditions can induce heritable genome changes in a specific gene(s) and (ii) the induced changes help adaptation to the environmental conditions (Fig. 1, upper panel). However, according to Darwin’s theory of evolution (1859) which is widely accepted, mutations occur at random, and environmental conditions act as a selective force on the resulting phenotype (Fig. 1, lower panel). This selection process promotes the fixation of adaptive mutations under appropriate conditions. Thus, Darwin’s theory does not demand a mechanism in which a specific mutation occurs in a specific gene that is required for adaptation.

The strongly negative view that exists today toward Lamarckian inheritance compared with the accepted Darwinian view may be partly due to an unfortunate history. In the 1880s, the German biologist August Weismann demonstrated that cutting off the tails of rats did not produce any tail-less pups (Weismann 1889), which gave the concept of the ‘inheritance of acquired characteristics’ a seriously negative image. However, at that time, people were not particularly interested in Lamarck’s concept of the inheritance of adapted (useful) changes, rather than senseless traits.

At the beginning of the 20th century, the Austrian biologist Paul Kammerer tried to prove the ‘inheritance of acquired characteristics’ by a series of experiments using amphibians (Kammerer 1924) (Table 1). The midwife toad mates on land and the males incubate the eggs on their legs, which is very unusual for an amphibian and is the origin of the name ‘midwife’. When Kammerer housed midwife toads in dry conditions at high temperatures, the animals stayed in a bowl of water, mated, and deposited the eggs in the bowl. Once the pups grew up, they mated and deposited their eggs in water, and the males no longer ‘midwifed’ the eggs. Kammerer reported that water-mating male toads had black ‘nuptial’ pads, which midwife toads do not. However, it was found that black ink had been injected into a toad, generating artificial nuptial pads. Just after the Nature paper (Noble 1926) that suggested that Kammerer had committed fraud, he killed himself.

**Table 1:** Evidence of inheritance and memory of stress or environmental effect

Stress or condition	Organism	Phenotype	Maintenance manner*	References
Arid condition	Midwife toad	Mating in water (?)	G (?)	Kammerer (1924); Pennisi (2009)
Nutrition limitation	Human	Increased lifestyle-related diseases	NG	Barker (1998)
Food supply	Human	Mortality	G	Pembrey *et al.* (2006)
Methyl donor supplement	Mouse	Decreased *Agouti* expression	G (?)/NG	Waterland & Jirtle (2003)
High-fat diet	Rat	β-cell dysfunction	G	Ng *et al.* (2010)
Low protein diet	Mouse	Metabolic gene expression	G	Carone *et al.* (2010)
UV light	*Arabidopsis thaliana*	Increased recombination	G	Molinier *et al.* (2006)
X-ray	Mouse	Elevated mutation rate	G	Barber *et al.* (2002)
Vinclozolin	Human	Decreased maternal fertility	G	Anway *et al.* (2005)
Reduced maternal care	Rat	Fearful phenotype	NG	Weaver *et al.* (2004)
Heat and osmotic stress	*Drosophila melanogaster*	Disrupted heterochromatin	G	Seong *et al.* (2011)

* G, gametic phenomena. Inheritance over generation.

NG, nongametic phenomena, which persist into adulthood. (?), not completely proven.

Recently, however, it has been suggested that Kammerer might have used ink to mark a color change that he actually observed and that he was in fact the discoverer of an epigenetic phenomenon (Pennisi 2009; Vargas 2009), although the opposing opinion still exists (Gliboff 2010). The truth remains unclear; but, following Kammerer’s experiments, it was difficult to improve the reputation of Lamarckian inheritance. In addition, at that time, the Bolshevik leaders of the new Soviet Union were pleased to accept Lamarck’s theories and Kammerer’s ideas of fast and planned improvements to nature, including human nature. Under the leadership of the agronomist Trofim Lysenko, Lamarck’s ideas were altered and used to support his own unscientific theories. Some of Lysenko’s claimed agricultural improvements did have a scientific basis, such as the ‘vernalization’ of wheat, although this process had been known about for decades. Unfortunately, Lysenko was extremely popular, and almost all meaningful biological research in the Soviet Union was blocked for decades (Soyfer 2001). Lysenko’s actions made Lamarckian inheritance theory even more unacceptable to most scientists.

Recently, data on the inheritance of stress effect have been accumulating, and some mechanistic insights, such as epigenetic regulation, have also been reported. In particular, the finding that stress-induced epigenome changes can be inherited may affect the modern view of Lamarckian inheritance. This review summarizes the current data on the inheritance of stress effect and possible mechanisms involved in this process. In particular, we focus on the stress-induced epigenome changes mediated by the ATF-2 family of transcription factors.

## Epigenetic regulation of heterochromatin

Epigenetics is a genetic phenomenon that depends on the chemical modification of DNA or histones, but not on the DNA sequence (Goldberg *et al.* 2007). Therefore, it is a form of inheritance that is independent of classical Mendelian inheritance. A typical example of epigenetics is DNA methylation, which is important for parent-of-origin-specific gene expression, also known as genome imprinting (Reik & Walter 2001). Imprinted alleles are silenced, and the genes are either expressed only from the nonimprinted allele inherited from the mother (e.g., H19 or CDKN1C) or the nonimprinted allele inherited from the father (e.g., IGF-2). Histone modifications are another important form of epigenetics. During the past decade, various histone modifications associated with transcriptional activation and silencing have been identified (Kouzarides 2007). Heterochromatin in close proximity to the centromere or telomere is transcriptionally inactive and is enriched in methylated DNA and trimethylation at histone H3-K9 (H3K9me3) (Grewal & Moazed 2003). Heterochromatin is important for the maintenance of chromosome structure and gene silencing, which is maintained not only during mitosis but also during meiosis. Among histone H3-K9 trimethyl transferases that bear the SET domain, such as SUV39h1, SUV39h2, and ESET (also called SETDB1), SUV39h1 mainly contributes to heterochromatin formation in somatic cells (Martin & Zhang 2005). Heterochromatin protein 1 (HP1) binds to H3K9me3 via its chromo domain, resulting in a tight chromatin structure.

Research into heterochromatin formation was started by using the fission yeast *Saccharomyces pombe*. The RNA interference (RNAi) machinery is required for the formation and maintenance of heterochromatin (Volpe *et al.* 2002). RNAi is a posttranscriptional silencing mechanism in which double-stranded RNA triggers the degradation of its target mRNA. Mutations in the RNAi machinery in *S. pombe*, such as in Dicer (Dcr1), argonaute (Ago1), and RNA-directed RNA polymerase 1 (Rdp1), result in defects in heterochromatin formation (Grewal & Elgin 2007). Pericentric outer repeats are transcribed, and the resulting transcripts are processed into small RNAs by the RNAi machinery. The generated small RNAs recruit histone modifiers to the pericentric repeats to establish histone modifications, such as H3K9me3, and to initiate HP1 binding, which results in the establishment of transcriptional silencing.

## Evidence of inheritance and memory of various stresses

Living organisms are frequently exposed to various stresses, which can induce transgenerational modifications of the genome. Such modifications include DNA methylation and histone modification. These chemical modifications of DNA and histones, not DNA sequence change, are known as epigenetic change. Certain stresses, for example, environmental conditions, have been reported to create epigenetic ‘memories’, some of which are inherited (Table 1). Nutrition is now thought to be one of the more important environmental conditions capable of modulating the epigenome. In 1998, David Barker reported that a low birth weight increases the risk of lifestyle-related diseases, such as type 2 diabetes in adulthood (Barker 1998). Limited nutrition *in utero* may induce compensatory responses, which represent adaptations to prepare for postnatal life. Increased insulin resistance, a typical adaptation, allows energy conservation to give the offspring a better chance of survival under a poor nutritional environment. However, insulin resistance leads to a higher blood glucose level, which is problematic in the presence of a rich diet in adulthood. This hypothesis, known as the ‘thrifty phenotype’ hypothesis, is supported by evidence from the offspring of pregnant women exposed to the Dutch famine during World War II (Bateson 2001), who had impaired glucose tolerance in adulthood after prenatal starvation. Furthermore, some reports even suggest that epigenetic effects induced by nutritional conditions can be multigenerational in human populations. Records, which include yearly crop yields, from a population in Överkalix in Sweden revealed a link between the nutritional status of grandparents and the mortality risk ratios of their grandchildren (Pembrey *et al.* 2006). For example, if the paternal grandfather enjoyed a period of high food availability between the ages of 9–12, his male grandchildren showed reduced longevity.

Some reports suggest that nutritional conditions regulate the methylation status of DNA. The *agouti viable yellow* (*A*^*vy*^) allele contains intracisternal A particle (IAP) retrotransposons that influence the expression of linked genes regulating coat color. This influence is dependent on the methylation status of the IAP long terminal repeat (LTR) promoter. Methyl donor supplementation of pregnant females via folic acid, vitamin B12, choline, or betaine altered the coat color in the offspring toward the repressed state (pseudoagouti) by causing hypermethylation of the *A*^*vy*^ allele (Waterland & Jirtle 2003). However, two research groups have reported conflicting answers to the question of whether this effect is inherited by the next generation (Cropley *et al.* 2006; Waterland *et al.* 2007).

Very recently, transgenerational inheritance of the nutritional effect was studied more precisely using DNA microarray analysis. When male rats were fed on a high-fat diet, their adult female offspring had normal body fat, but showed a pancreatic β-cell impairment and altered expression of 642 genes that are involved in pathways controlling insulin regulation and glucose metabolism (Ng *et al.* 2010). DNA hypomethylation of a cytosine residue in the 5′ region of the *Il13ra2* gene encoding IL13 receptor subunit α2, which showed the greatest alteration in expression, was exhibited in these females. In another report, when male mice were fed on a low-protein diet, the offspring of both sexes showed altered expression of genes involved in fat and cholesterol biosynthesis (Carone *et al.* 2010). Numerous, moderate changes in DNA methylation were detected at many sites in the livers of the offspring.

Environmental stress may also modulate the epigenome, which might affect the evolution of the organism in question. For example, ultraviolet (UV) light stress leads to increased homologous recombination in *Arabidopsis thaliana,* even in unstressed progeny, for up to four generations (Molinier *et al.* 2006). This phenomenon seems to be epigenetic because the whole population showed changes in each generation. In addition, the effect acted *in trans* on a reporter transgene derived from an untreated parent and could be transmitted both maternally and paternally.

Similar transgenerational epigenetic effects induced by ionizing radiation have also been observed in mice. X-ray exposure of *F*_0_ mice increased the mutation rate at expanded simple tandem repeat (ESTR) loci in the *F*_1_ and *F*_2_ generations (Barber *et al.* 2002). Mutation rates were maintained at high levels in the *F*_1_ and *F*_2_ germ-lines, indicating the presence of an epigenetic mechanism. The fungicide vinclozolin is widely sprayed on vineyards and remains in the environment. Rats exposed to vinclozolin at the time of gonadal sex determination showed fertility defects in male offspring, which were transmitted for at least three generations (Anway *et al.* 2005). Increased DNA methylation was detected in sperm from vinclozolin-exposed males, and these methylation patterns were inherited.

The third important stress factor that may affect the epigenome is psychological stress. Postnatal maternal licking/grooming and arched-back nursing (LG-ABN) is a typical behavior seen in rat mothers when taking care of pups. Low levels of LG-ABN cause offspring to be more fearful, a behavior that persists into adulthood. The reduced fearfulness of high LG-ABN rat offspring is the result of an increase in the number of glucocorticoid receptors in the hippocampus. Low LG-ABN mothering results in a low level of glucocorticoid receptors (GR) in the hippocampus, which is associated with DNA hypermethylation and decreased histone acetylation of the GR gene (Weaver *et al.* 2004).

## ATF-2 is a target of the stress-activated protein kinase p38

**Figure 2:**
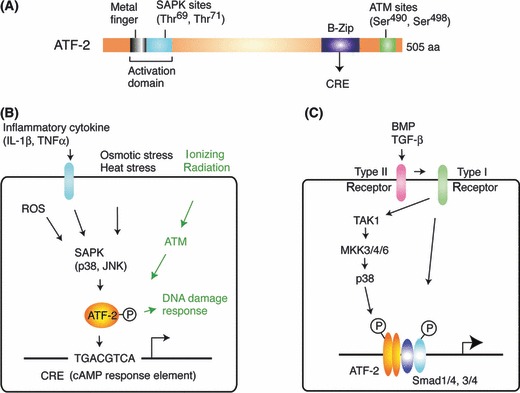
ATF-2 is a nuclear target of p38/Jun N-terminal protein kinase (JNK). (A) Domain structure of ATF-2. ATF-2 contains a B-ZIP-type DNA-binding domain and a *trans*activation domain, which consists of a metal finger structure and SAPK phosphorylation sites. (B) ATF-2 is activated by SAPKs in response to various stresses, including inflammatory cytokines, environmental stresses, and DNA damage. These stresses induce the phosphorylation of ATF-2 via SAPKs, which then activates the transcription of target genes. DNA double-strand breaks induce the phosphorylation of ATF-2 via ATM, which then participates in DNA repair. (C) Role of ATF-2 in the bone morphogenetic protein (BMP)/TGF-β signaling pathway. ATF-2 is phosphorylated via the TAK1-p38 pathway in response to BMP/TGF-β, although BMP/TGF-β induces the phosphorylation of Smad1 or Smad3, which leads to the nuclear entry of the Smad1/4 or Smad3/4 complex. In the nucleus, ATF-2 interacts with the Smad complex and synergistically activates transcription.

Recently, it was shown that the ATF-2 family of transcription factors is involved in stress-induced epigenome changes (Maekawa *et al.* 2010b; Seong *et al.* 2011), and in this review, we summarize the basic characteristics of this transcription factor family. Human ATF-2 (originally called CRE-BP1) was first identified as a factor that binds to the cyclic AMP response element (CRE: 5′-TGACGTCA-3′) (Maekawa *et al.* 1989) and was then found to be identical to one of the ATF/CREB family of transcription factors, which possess a B-ZIP DNA-binding domain (Hai *et al.* 1989). ATF-2 forms a homodimer as well as a heterodimer with c-Jun and activates a group of target genes (Hai & Curran 1991; Matsuda *et al.* 1991). The *trans*activation domain of vertebrate ATF-2 consists of a zinc finger motif and phosphorylation sites (Thr^69^ and Thr^71^) for stress-activated protein kinases (SAPKs) such as p38 and Jun N-terminal protein kinase (JNK) (Fig. 2A) (Gupta *et al.* 1995; Nagadoi *et al.* 1999). SAPKs are activated by inflammatory cytokines, which are induced by various stresses such as pathogen infection and psychological stress. They are also activated by environmental stresses such as heat stress, osmotic stress, and hypoxia, as well as reactive oxygen species (ROS), which are correlated with metabolism (Chang & Karin 2001; Craig *et al.* 2004). In response to these various stresses, p38 and JNK phosphorylate ATF-2 directly and enhance its *trans*activating capacity (Fig. 2B) (van Dam *et al.* 1997; Livingstone *et al.* 1997; Brinkman *et al.* 1999). ATF-2 is also phosphorylated by the protein kinase, ATM, at Ser^490^ and Ser^498^ after ionizing radiation (Fig. 2B) (Bhoumik *et al.* 2005). The phosphorylation of ATF-2 by ATM results in its rapid localization at ionizing radiation-induced foci where it enhances the recruitment of the double-strand break repair gene product, Mre11, indicating a role for ATF-2 in DNA damage response that is independent from its transcriptional activity.

As p38 is not only involved in stress responses but also a member of a signaling pathway that responds to growth factors, such as the bone morphogenetic protein (BMP)/TGF-β (transforming growth factor β) super family, ATF-2 also plays a role in signaling pathways. The BMP/TGF-β-TAK1 (TGF-β-activating kinase 1) pathway induces the phosphorylation of ATF-2 by p38 (Fig. 2C) (Sano *et al.* 1999; Monzen *et al.* 2001). However, BMP/TGF-β also induces the phosphorylation of the transcription factor Smad3, which results in the nuclear entry of the Smad3/Smad4 complex (Heldin *et al.* 1997). In the nucleus, phosphorylated ATF-2 (P-ATF-2) interacts with Smad3/Smad4 to activate transcription synergistically in response to BMP/TGF-β stimulation (Sano *et al.* 1999). Furthermore, insulin, epidermal growth factor, and serum also activate ATF-2 via two pathways: the Raf–MEK–ERK and the Ral–RalGDS–Src–p38 pathways (Ouwens *et al.* 2002). Cooperation between ERK and p38 is essential for ATF-2 activation by these growth factors.

**Figure 3:**
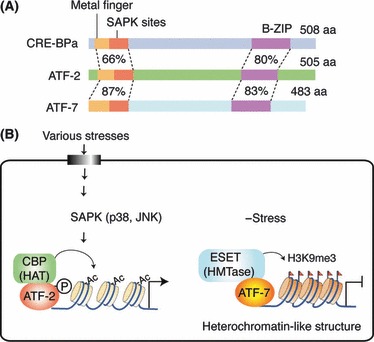
Distinct functions of three members of the vertebrate ATF-2 family. (A) Homology between three members of the vertebrate ATF-2 family. (B) Two different functions of the ATF-2 transcription factor family. ATF-2 activates transcription in response to various stresses by interaction with the coactivator, CBP, which induces the acetylation of histones. In the absence of stress, ATF-7 silences transcription via interaction with the histone H3-K9 trimethyltransferase ESET (SETDB1), which forms a heterochromatin-like structure.

The vertebrate ATF-2 subfamily contains two other members, CRE-BPa (also known as CREB5) and ATF-7 (originally known as ATF-a) in addition to ATF-2 (Gaire *et al.* 1990; Nomura *et al.* 1993). Each of these proteins contains a *trans*-activation domain consisting of a metal finger structure and SAPK phosphorylation sites, and a B-ZIP-type DNA-binding domain (Fig. 3A) and is expressed in various tissues and cells (Takeda *et al.* 1991; Nomura *et al.* 1993; Goetz *et al.* 1996). ATF-2 is phosphorylated either by p38 or JNK, although ATF-7 is phosphorylated by p38, but not by JNK (De Graeve *et al.* 1999). In addition, both ATF-2 and CRE-BPa activate transcription from CRE promoters via interactions with coactivator CBP (Sano *et al.* 1998), although ATF-7 represses transcription. ATF-7 binds to mouse ATFa-associated modulator (mAM), which is a component of the ESET complex (De Graeve *et al.* 2000; Wang *et al.* 2003). ESET is a histone methyltransferase (HMTase) that converts lysine nine of histone H3 (H3-K9) from the dimethyl to the trimethyl form, and ATF-7 is thought to support gene silencing by inducing histone H3-K9 trimethylation (H3K9me3). These data suggest that ATF-2 and CRE-BPa induce the transcription of a group of target genes in response to various stresses; however, ATF-7 silences transcription in the absence of stress (Fig. 3B).

## Role of the ATF-2 family of transcription factors in development and cancer

The physiological role of the ATF-2 family of transcription factors has been examined using knockout mice (Table 2). *Atf-2* null mutant mice die immediately after birth because of severe respiratory defects with lungs filled with meconium (Maekawa *et al.* 1999). This phenotype is similar to the severe type of human meconium aspiration syndrome (MAS). The mutant placenta shows a reduced number of cytotrophoblast cells, which may lead to an insufficient supply of oxygen to the embryo before birth, followed by strong gasping respiration during labor with consequent aspiration of the amniotic fluid containing meconium. The platelet-derived growth factor receptor α (PDGF receptor α) gene, which plays an important role in the proliferation of trophoblasts, was found to have reduced levels of expression in the mutant cytotrophoblasts. Furthermore, co-transfection experiments demonstrated that ATF-2 binds directly to the 5′ region of the PDGF receptor α gene and activates transcription. These data confirmed that the PDGF receptor α gene is regulated directly by ATF-2, which may be activated in response to hypoxic stress during placental development. Hypoxic stress occurs during normal placental development (Giaccia *et al.* 2004), and the hypoxia-responsive transcription factors HIF-1α and HIF-2α are required for the differentiation of trophoblast cells in the placenta (Cowden Dahl *et al.* 2005). As ATF-2 is also activated by hypoxia (Maekawa *et al.* 2008), it may activate a group of target genes, including the PDGF receptor α, in response to hypoxic stress. Hypomorphic *Atf-2* mutant mice, which expressed an ATF-2 fragment, showed decreased postnatal viability and growth, with defects in endochondral ossification and in the neuronal system (Reimold *et al.* 1996), suggesting that ATF-2 is also required for skeletal and central nervous system development.

**Table 2:** Physiological role of ATF-2 family transcription factors

Member of ATF-2 family	Mutant	Phenotype	References
Mouse ATF-2	Null mutant	Neonatal lethal/placental defect	Maekawa *et al.* (1999)
Mouse ATF-2	Heterozygous mutant	Mammary tumors	Maekawa *et al.* (2007)
Mouse ATF-2	Keratinocyte-specific mutant	Skin tumor	Bhoumik *et al.* (2008)
Mouse ATF-2	Hypomorphic mutant	Chondrodysplasia and neurological abnormalities	Reimold *et al.* (1996)
Mouse CRE-BPa	Null mutant	Neonatal lethality/lung defect	Maekawa *et al.* (2010a)
Mouse ATF-2 and CRE-BPa	*trans*-Heterozygous mutant	Reduced white adipose tissue	Maekawa *et al.* (2010a)
Mouse ATF-2 and ATF-7	ATF-2 Ala and ATF-7 null mutant	Embryonic lethal/liver and heart defect	Breitwieser *et al.* (2007)
Mouse ATF-7	Null mutant	Abnormal behavior	Maekawa *et al.* (2010b)
*Drosophila melanogaster* ATF-2	PiggyBac insertion	Disruption of heterochromatin	Seong *et al.* (2011)
*D. melanogaster* ATF-2	Reduction in fat body	Reduced glyceroneogenesis	Okamura *et al.* (2007)
*D. melanogaster* ATF-2	Reduction in pacemaker neurons	Abnormality in sleep and locomotion	Shimizu *et al.* (2008)
*Caenorhabditis elegans* ATF-7	Null mutant	Abnormality in innate immunity	Shivers *et al.* (2010)

**Figure 4:**
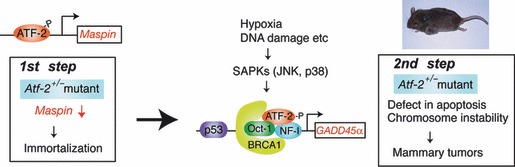
ATF-2 acts as a mammary tumor suppressor. (Left panel) In the *Atf-2* heterozygote, *Maspin* expression is reduced, which leads to the immortalization of cells. (Right panel) Immortalized cells are exposed to hypoxia or DNA damage. Although these stresses activate apoptosis by inducing the *Gadd45α* gene via ATF-2 in wild-type cells, *Atf-2* heterozygous cells escape this response, which leads to mammary tumors.

Heterozygous *Atf-2* mutant mice are highly prone to developing mammary tumors after long periods of latency because of dramatic reductions in the expression of *Maspin*, a mammary tumor suppressor gene, and *Gadd45α,* which is induced by hypoxic stress (Maekawa *et al.* 2007, 2008). ATF-2 regulates the transcription of *Maspin* via direct binding to the *Maspin* gene and *Gadd45α* transcription through interactions with two transcription factors, Oct-1 and NF-I, and the breast cancer tumor suppressor BRCA1 (Fig. 4). As *Maspin* enhances cellular sensitivity to apoptotic stimuli, decreased *Maspin* expression in *Atf-2* heterozygotes could contribute to immortalization by the abrogation of apoptosis. Once immortalized, *Atf-2*^*+/−*^ cells are less able to induce apoptosis in response to hypoxia, to which solid tumors such as mammary tumors are exposed, via the down-regulation of the apoptosis-related genes, including *Gadd45α.* Consistent with these data, the knockout of *Atf-2* in the basal layer of the epidermis resulted in an increase in the incidence and prevalence of papilloma development when subjected to a two-stage skin carcinogenesis protocol using DMBA/TPA (Bhoumik *et al.* 2008). The expression of presenilin1 was reduced, and the expression of its target genes, β-catenin and cyclin D1, was enhanced in the papillomas of the *Atf-2* mutant mice.

Like the *Atf-2* null mutant, *Cre-bpa* mutant mice die immediately after birth because of respiratory problems caused by a defect in lung development, although the *trans*heterozygote *Atf-2* and *Cre-bpa* mutants are lean and have reduced white adipose tissue (Maekawa *et al.* 2010a). ATF-2 and CRE-BPa are required for BMP-2- and p38-dependent induction of peroxisome proliferator-activated receptor γ2 (PPARγ2), a key transcription factor involved in mediating adipocyte differentiation. In another study, the combination of a mouse carrying mutations in the ATF-2 phosphorylation sites with an ATF-7 knockout mutant resulted in embryonic lethality with severe abnormalities in the developing liver and heart (Breitwieser *et al.* 2007). The mutant fetal liver showed high levels of apoptosis in developing hepatocytes and hematopoietic cells because of the loss of a negative feedback loop involving the ATF-2-dependent transcriptional activation of MAPK phosphatases.

*Drosophila melanogaster* has one ATF-2 homolog (dATF-2), which contains the sites phosphorylated by p38 but not by JNK (Sano *et al.* 2005). This characteristic is similar to vertebrate ATF-7, which suggests that dATF-2 plays two roles, one in gene silencing in the absence of stress, in a manner similar to vertebrate ATF-7, and the second as a transcriptional activator in response to stress, like the vertebrate homologs ATF-2/CRE-BPa. It has been shown that dATF-2 is present in the fat body, which is the fly equivalent of the mammalian liver and adipose tissue, where it plays an important role in the regulation of fat metabolism by activating the transcription of the phosphoenolpyruvate carboxykinase (PEPCK) gene (Okamura *et al.* 2007).

Phosphorylated dATF-2 is expressed in large ventral lateral neurons in the brain, the pacemaker neurons. The knockdown of dATF-2 in pacemaker neurons decreased sleep time, whereas ectopic expression of dATF-2 increased sleep time (Shimizu *et al.* 2008). The dATF-2 protein negatively regulated locomotor activity, and the degree of dATF-2 phosphorylation was enhanced by forced locomotion via the dp38 pathway. Thus, dATF-2 is activated by the locomotor, although it increases sleep, suggesting a role for dATF-2 as a regulatory protein that connects sleep with locomotion.

The ATF-7 homolog in *Caenorhabditis elegans* regulates innate immunity (Shivers *et al.* 2010). It functions as a repressor of p38-regulated genes and undergoes a switch to an activator on phosphorylation by p38. As p38 is involved in innate immunity in multiple species (Han *et al.* 1998; Kim *et al.* 2002; Akira *et al.* 2006), the ATF-2 family of transcription factors may regulate innate immunity in metazoans in a similar manner.

## Role of ATF-7 in stress-induced epigenome change

**Figure 5:**
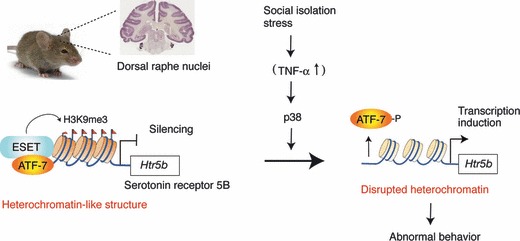
ATF-7 mediates *Htr5b* gene induction in response to social isolation stress. (Left) In dorsal raphe nuclei, ATF-7 binds to the *Htr5b* gene and silences its transcription by recruiting the H3-K9 trimethyltransferase ESET. (Right) Social isolation stress induces ATF-7 phosphorylation via p38, which then leads to the release of ATF-7 from the *Htr5b* gene promoter, resulting in *Htr5b* gene expression and the disruption of heterochromatin-like structures.

Atf-1, the fission yeast homolog of ATF-2, functions in heterochromatin nucleation independently of the RNAi machinery (Jia *et al.* 2004). Vertebrate ATF-2 also interacts with the histone variant macroH2A, which is enriched in the inactive X chromosome in female mammalian cells and functions to maintain gene silencing (Agelopoulos & Thanos 2006). Heterochromatin-like structure is also found at specific genes in euchromatin. Analysis of *Atf-7* knockout mice indicated a role for ATF-7 in gene silencing via the formation of a heterochromatin-like structure. *Atf-7*-deficient mice show abnormal behavior and increased 5-HT (serotonin) receptor 5B (*Htr5b*) expression in the dorsal raphe nuclei of the brain (Maekawa *et al.* 2010b). ATF-7 silences the transcription of *Htr5b* by binding directly to its 5′ regulatory region and recruits histone H3K9me3 via the ESET HMTase (Fig. 5, left). It is well known that wild-type mice reared in isolation show abnormal behavior (Rodgers & Cole 1993), and keeping one mouse alone in the cage induces strong social stress; thus, four to five mice are usually reared together in one cage. The abnormal behavior seen in *Atf-7*-deficient mice partly resembled that of wild-type mice reared in isolation, suggesting that social isolation stress disrupts ATF-7-dependent gene silencing. In fact, in response to isolation stress, it was found that ATF-7 is phosphorylated in the dorsal raphe nucleus and is then released from the *Htr5b* promoter, leading to the up-regulation of *Htr5b* (Fig. 5, right). The mechanism by which isolation stress induces phosphorylation of ATF-7 is currently unknown. We observed that the level of phosphorylated p38 increases in dorsal raphe nuclei after social isolation stress. Psychological stresses such as isolation may affect the activities of various kinases in the brain, such as cAMP-dependent protein kinase, through hormonal regulation (McGaugh & Roozendaal 2002). So far, however, only p38 has been shown to phosphorylate ATF-7 at Thr-51, which suggests that p38, but not other kinase(s), phosphorylates ATF-7 in dorsal raphe nuclei. One possible mechanism of p38 activation in the brain is an increase in the inflammatory cytokines by social isolation stress. Many reports have indicated that various psychological stresses induce inflammatory cytokines in peripheral tissues at levels that correlate with some mental diseases (Connor & Leonard 1998; Black 2002). Especially, the level of TNF-α is increased by social isolation stress (Wu *et al.* 1999). These cytokines may move into the brain, where they could activate the p38-ATF-7 pathway. However, we cannot exclude other possibilities, such as p38 activation via the adrenergic receptor-dependent pathway, which is modulated by psychological stress (Bierhaus *et al.* 2003). Thus, although further analyses are needed to clarify the mechanism, the results suggest that ATF-7 may play a critical role in epigenome change induced by social isolation stress.

## Inheritance of dATF-2-dependent, stress-induced epigenome change

**Figure 6:**
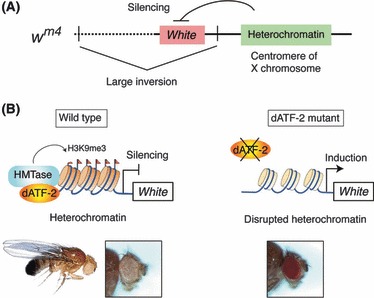
The dATF-2 protein is required for heterochromatin formation in *Drosophila*. (A) In the *w*^*m4*^ mutant line, the *white* gene is located close to centromeric heterochromatin on the X chromosome because of a large inversion. (B, left) In *w*^*m4*^, dATF-2 maintains the H3K9me3 status of heterochromatin, which causes *white* gene silencing and a mottled-eye phenotype. (B right) In the *w*^*m4*^ mutant carrying a *dATF-2* mutation, the degree of H3K9me3 in heterochromatin is reduced, resulting in heterochromatin disruption and *white* gene induction.

In *D. melanogaster*, the heterochromatin-dependent silencing (PEV) of *white* marker gene expression has been widely used to study the regulators of heterochromatin (Henikoff 1990). In the *w*^*m4*^ line, a large inversion places the *white* gene close to the centromeric heterochromatin on the X chromosome (Fig. 6A). Because of heterochromatin-mediated silencing, *white* gene expression (which is required for a red eye phenotype) is silenced, and the *w*^*m4*^ line shows a mottled (red and white)-eye phenotype (Fig. 6B, left). In contrast, when *w*^*m4*^ was combined with a *dATF-2* mutation, *white* silencing was almost completely abrogated, and the combined mutant fly had red eyes, indicating that dATF-2 is needed for heterochromatin formation (Fig. 6B, right) (Seong *et al.* 2011). Heterochromatin formation can be divided into two stages, establishment and maintenance (Hall *et al.* 2002), and dATF-2 contributes not only to the establishment of heterochromatin during early embryogenesis but also to the maintenance of heterochromatin in later stages. As in yeast, dATF-2 contributes to heterochromatin formation and is independent of the RNAi machinery.

**Figure 7:**
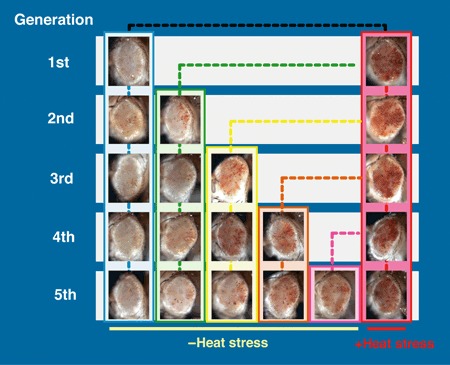
Inheritance of stress-induced epigenome change over multiple generations. (Green line and box) When flies were exposed to HS stress at the G1 generation only, its effect, as shown by the up-regulation of the red eye pigment, was transmitted only to G2-generation flies but not to successive generations. (Yellow line and box) When flies were exposed to HS stress at G1 and G2 generations, its effects were seen in the successive G3, G4, and G5 generations.

When *w*^*m4*^ flies were exposed to heat shock (HS) stress (37°C for 1 h) during early embryogenesis, *white* gene silencing was most effectively disrupted, suggesting that heterochromatin is more sensitive to stress during the establishment stage than at the maintenance stage (Fig. 7, first generation) (Seong *et al.* 2011). However, osmotic stress, which induces dATF-2 phosphorylation more efficiently than HS stress, was also capable of disrupting heterochromatin during the larval stage, indicating that strong stress may disrupt heterochromatin even during the maintenance stage. In response to HS or osmotic stress, dATF-2 is phosphorylated via the Mekk1-p38 pathway, leading to its release from heterochromatin and a reduction in the degree of H3K9me3.

When flies, which were exposed to HS during early embryogenesis or osmotic stress at a later stage, were mated with nonstressed flies, the progeny also showed partly disrupted heterochromatin (Fig. 7, green line and box) (Seong *et al.* 2011). This stress-induced epigenome change was transmitted to the next generation either maternally or paternally. In the case of maternal transmission, the *white* gene on the X chromosome in the progeny is always derived from the nonstressed female, indicating that the stress-induced epigenome change is inherited in a non-Mendelian fashion. This may occur by some form of transcommunication between chromosomes such as paramutation (Chandler 2007). When first-generation flies were exposed to HS stress, the disrupted heterochromatin state was inherited by the second generation, but not by successive generations (Fig. 7, green line and box), indicating that the new HS-induced epigenetic state is unstable. When flies were exposed to HS stress at the first and second generations, the disrupted heterochromatin state was transmitted to the successive three generations, although the effect of HS stress was gradually weakened (Fig. 7, yellow line and box). Thus, exposure to stress over multiple generations stabilizes the degree of epigenetic change and prolongs the period of inheritance.

## Future perspectives

### Target genes of the ATF-2 transcription factor family

Putative dATF-2 target genes were identified on the basis of the following criteria: HS induced the expression of certain genes, and this up-regulation was inherited by the next generation in wild-type *Drosophila* but not in *dAtf-2* mutant flies (Seong *et al.* 2011). As described previously, members of the ATF-2 family of transcription factors are activated by p38 in response to various stresses, including environmental stress and the production of ROS, which is correlated with nutrition, psychological stress, and pathogen infection. Therefore, it is important to identify those genes that are silenced by dATF-2/ATF-7 and induced by those stresses. Furthermore, it would be interesting to determine which of the target genes show inheritance of stress-induced expression.

Recently, two groups have identified genes in which changes induced by nutritional conditions were transmitted to the next generation (Carone *et al.* 2010; Ng *et al.* 2010), and it is possible that some of these genes might be regulated by ATF-7. The identification and analysis of the regulatory mechanisms controlling such genes might provide clues that would help to understand the mechanism behind Barker’s ‘thrifty phenotype’ hypothesis.

To date, there have been no reported data indicating the inheritance of psychological stress-induced gene expression. Social isolation stress was shown to induce *Htr5b* expression, which is normally silenced by ATF-7 (Maekawa *et al.* 2010b); however, we were unable to examine its inheritance, as mice exposed to social isolation stress had a defect in sexual behavior (unpublished data). It is vital to determine whether psychological stress-induced gene expression is inherited because it could help to understand the inheritance of mental illness.

If the dATF-2/ATF-7 transcription factors are also involved in regulating the epigenome state of innate immunity-related genes, such phenomena might be correlated with the inheritance of innate immunity. There is no evidence that immune memory is inherited, and it is believed to last for only one generation. Long-term immunity is acquired via the induction of memory T and B cells, after infection, although it is widely believed that innate immunity has no memory function. However, recent evidence suggests the presence of memory-like phenomena in plants and invertebrates (Netea *et al.* 2011). Further analysis of the roles of dATF-2/ATF-7 could be the key to understand innate immunity memory.

### Mechanism of stress-induced epigenome inheritance

To transmit a stress effect to the next generation, the stress-induced epigenome change should occur in the gamete. The dATF-2 protein has been shown to exist in *Drosophila* germ cells (Seong *et al.* 2011), and ATF-7 is expressed in the germ cells of mouse testis (unpublished data). Various stresses may induce dATF-2/ATF-7-dependent epigenome changes in germ cells, and these may be transmitted to the next generation. The germ cell genome undergoes a drastic reprogramming process during differentiation, including histone replacement by protamines and the erasure of DNA methylation. The dATF-2/ATF-7 transcription factors are required for the formation and maintenance of centromeric heterochromatin and for the maintenance of heterochromatin-like DNA in euchromatin regions. DNA methylation and histone H3-K9 trimethylation are two major marks that identify repressive heterochromatin structure, although histone replacement by protamines during mammalian spermatogenesis erases the epigenetic marks on histones. However, in human and mouse sperm, approximately 15% and 2%, respectively, of the genome remains nucleosome bound (Bench *et al.* 1996), and these nucleosome-bound regions are enriched in heterochromatin (van der Heijden *et al.* 2006). DNA methylation in centromeric heterochromatin is also resistant to postfertilization demethylation (Rougier *et al.* 1998). Thus, it appears that heterochromatin structure is somehow maintained during the reprogramming of germ cells, although the precise mechanism remains unknown.

It is possible that once heterochromatin is partly disrupted by stress via dATF-2/ATF-7, this disrupted state could be transmitted to the next generation. The role played by RNA in heterochromatin formation is well known, and a genetic screen to identify modifiers of heterochromatin and paramutation led to the isolation of DNA-dependent RNA polymerase and RNA-dependent RNA polymerase, respectively (Kato *et al.* 2005; Alleman *et al.* 2006). The latter might be required to establish and maintain the heritable chromatin state associated with paramutation. It has been shown that the suppression of target genes by siRNAs in the maternal germ-line of *C. elegans* is transmitted for up to three generations (Grishok *et al.* 2000). In addition, a mouse white tail phenotype, associated with the *Kit* mutation, has been transmitted to wild-type mice via abnormal *Kit* RNA from sperm (Rassoulzadegan *et al.* 2006). These data suggest the possibility that transcript(s) from heterochromatin contribute to the formation of repressive chromatin structure, which depends on the primary DNA sequence. If such a mechanism functions during germ cell development and/or the establishment of heterochromatin in early embryogenesis, it should be able to repair heterochromatin partly disrupted by stress. This idea is consistent with the observation that stress-induced epigenome change is not a stable trait and cannot be transmitted over many generations (Seong *et al.* 2011) because such a repair system depends only on the underlying DNA sequence. Therefore, the stability of the altered epigenome status would depend on the degree of disruption of heterochromatin.

### Why has ATF-2 been selected as the regulator of heterochromatin?

Why have members of the ATF-2 family, rather than any other family of transcription factors, been selected during evolution as regulators of heterochromatin? A common feature of heterochromatin is the presence of various transposable elements (TEs) of different origins. In *Drosophila*, centric heterochromatin is considered to be a grave for dead transposable elements because of the low frequency of recombination in this region. There are thousands of different TE families, which constitute 80% or more of the total genomic DNA in plants and 3%–45% in metazoans (Hua-Van *et al.* 2005). Mutations caused by the insertion of TEs result in a great diversity of phenotypes (van de Lagemaat *et al.* 2003). In various organisms, the jumping of some TEs can be induced by stress; for example, McClintock demonstrated the activation of TEs in maize under stress and the importance of this stress-induced TE mobility for the emergence of resistance phenotypes (1984). However, the mechanism controlling the stress-induced jumping of TEs remains unknown. One possibility might be that the ATF-2 family of transcription factors is involved in the stress-dependent activation of TEs, which has helped to generate biological diversity in response to stresses. As heterochromatin has been derived from TEs, the role of ATF-2 as a regulator of TEs might be still remained in heterochromatin.

There have been some reports suggesting that other signaling pathways regulate the epigenetic state. Activation of the JAK-STAT pathway disrupts heterochromatin to cause blood tumor formation (Shi *et al.* 2006), and unphosphorylated STAT stabilizes heterochromatin by binding to HP1 (Shi *et al.* 2008). In addition to heterochromatin, polycomb target genes have a tight, repressive chromatin structure enriched in histone H3-K27me3. The methylation of H3-K27 can be modulated by the Nodal-Smads2/3 signaling pathway, which recruits the histone demethylase Jmjd3 (Dahle *et al.* 2010). Although it is unknown whether epigenetic changes in one generation can be transmitted to the next generation, transcription factors other than ATF-2 might also be involved in epigenetic changes.

### Is epigenome inheritance correlated with Lamarckian inheritance?

The epigenome changes induced by stress are not stable traits that can be transmitted over many generations, which suggests that stress-induced epigenome change does not correspond to the environment-dependent changes of characteristics proposed by Lamarck, which are transmitted over many generations. In theory, however, disruption of the epigenome status could lead to a subsequent mutagenic event by altering cytosine methylation, which could lead to a change in the frequency of C/G-to-T/A transitions. Furthermore, in some cases, an open chromatin state induced by transcription can increase the mutation frequency, as seen when somatic hypermutation in the heavy chain locus correlates with transcription (Yoshikawa *et al.* 2002). Therefore, examining whether stress-induced epigenome change can lead to stable changes in the DNA sequence will be an important step in understanding the role of epigenetic change in Lamarckian inheritance.
